# The Outbreak of SARS-CoV-2 in a Medical Ward and Its Containment: An Experience From a Tertiary Care Centre in North India

**DOI:** 10.7759/cureus.41795

**Published:** 2023-07-12

**Authors:** Nazneen N Begam, Saikat Mondal, Eram Afroz, Arghya Das, Ashutosh Biswas, Naveet Wig

**Affiliations:** 1 Infectious Diseases, AIl India Institute of Medical Sciences, New Delhi, IND; 2 Internal Medicine, All India Institute of Medical Sciences, Kalyani, IND; 3 Internal Medicine, Dr. Ram Manohar Lohia (RML) Institute of Medical Sciences, Lucknow, IND; 4 Microbiology, All India Institute of Medical Sciences, Madurai, IND; 5 Internal Medicine, All India Institute of Medical Sciences, Bhubaneshwar, IND; 6 Internal Medicine, All India Institute of Medical Sciences, New Delhi, IND

**Keywords:** healthcare worker safety, investigation of outbreak, communicable disease control, hospital outbreak, sars cov2

## Abstract

Background

Periodic outbreaks of SARS-CoV-2 in hospital settings amidst the recent pandemic are well known. However, the timely control of such outbreaks was key to preventing morbidity among vulnerable patients as well as reducing sickness absenteeism among healthcare workers. This is the first study of its kind in India.

Methods

An outbreak investigation was conducted between June 12 and July 18, 2020, at the All India Institute of Medical Sciences, New Delhi, India, during the first wave of COVID-19.

Results

A total of 27 individuals were infected during this time, including people visiting the hospital and healthcare workers. A thorough investigation led us to the epidemiological link between cases and allowed us to bring reforms to the existing hospital policy of screening and admission of COVID-19 patients and those suspected to have the infection. This experience helped us avoid future outbreaks during the second wave of COVID-19 in our hospital.

Conclusion

The SARS-CoV-2 virus is highly transmissible, especially in hospital settings due to the high burden of patients and close proximity between patients. Timely intervention is the key to effective control of hospital outbreaks, as it can avoid morbidity in patients and reduce sickness absenteeism among healthcare workers.

## Introduction

The SARS-CoV-2 virus took a toll on human existence right from its emergence. The virus spread all over the world and the WHO declared it the COVID-19 pandemic on 11th March 2020 [1]. Subsequently, various global health bodies worked tirelessly to find appropriate measures to control the disease. Several guidelines on infection prevention and control were published by different organizations such as the WHO and the CDC to tackle the virus in the community as well as hospital settings. Despite following the various preventive strategies (viz. contact, droplet, and airborne precautions) outlined in these guidelines, SARS-CoV-2 was reported to cause massive outbreaks in healthcare settings of different countries including China, the United Kingdom, Canada, Thailand, and the United States [2-4]. There was, however, no report of any such healthcare-associated or hospital outbreak from India. Since the outset of the COVID-19 pandemic, it became evident that hospitals and healthcare facilities were important settings for viral transmission. Healthcare workers were at risk owing to multiple reasons. Hospital transmission of the virus was unlikely if proper prevention and control measures were adopted and followed. Detailed studies of hospital outbreaks gave us clues to identify residual systemic weaknesses that led to the occurrence of multiple hospital outbreaks despite the preventive measures [5]. We investigated an outbreak of COVID-19 in a hospital ward and established the epidemiological link between the cases.

## Materials and methods

Outbreak setting and investigation period

**Figure 1:**
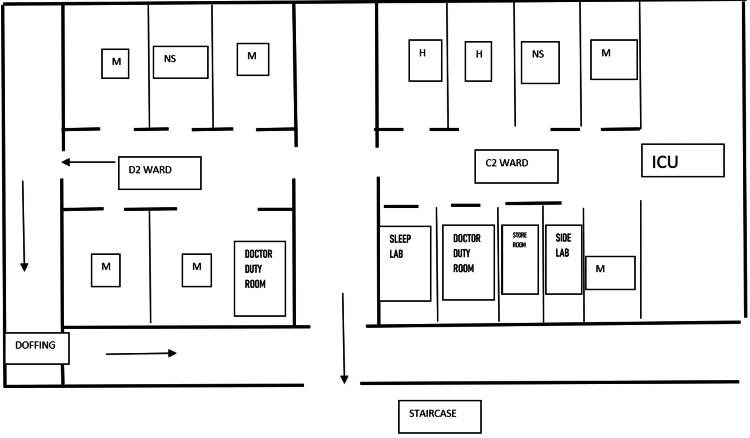
Floor map of the medicine and hematology ward M: Medicine cubicle, H: Hematology cubicle, NS: Nursing station

An outbreak investigation was carried out in the medical ward of a tertiary care center and teaching hospital in India. A total of 67 patients were admitted to the ward under the Department of General Medicine and the Department of Hematology. The floor map of the ward is depicted in Figure 1. Since the beginning of the COVID-19 pandemic in 2020, the institutional policy has been to admit COVID-19 suspect patients in a separate COVID-19 ward, and upon a negative test result, patients are shifted to their respective specialty wards. The investigation was carried out between June 12 and July 18, 2020.

Confirmation of the diagnosis

Nasopharyngeal and oropharyngeal swab specimens were collected in the viral transport medium (VTM) from patients and healthcare personnel (HCP) with symptoms suggestive of COVID-19 (viz., fever or chills, cough, shortness of breath or difficulty breathing, muscle or body aches, headache, new loss of taste or smell, sore throat, etc.). These were tested with real-time reverse transcriptase polymerase chain reaction (rRT-PCR) kits supplied by the Indian Council of Medical Research.

Case definitions

The following case definitions were used for patients involved in the outbreak: a *definite hospital-acquired infection* was defined as rRT-PCR positive after 14 days of admission and a negative rRT-PCR report at the time of admission; a *probable hospital-acquired infection* was defined as rRT-PCR positive between 7 to 14 days of admission; a *possible hospital-acquired infection* was defined as rRT-PCR positive within 7 days of admission.

Line listing of cases

Data on admitted patients were collected from the individual patient data sheets and files. Data from the infected HCP were collected telephonically. The demographic, clinical, and exposure information of all cases were documented for the line listing.

Annotation of the cases and their epidemiological link

Patients were marked as ‘P’ followed by a numerical number based on their sequence of involvement in the outbreak. Similarly, the HCP were marked by the initials of their designation followed by a numerical number. The epidemiological link was established based on the timing of exposure, the appearance of symptoms, and laboratory confirmation of the infection.

Data analysis, presentation, and calculation of basic reproduction number (R0)

The data were analyzed temporally and presented with graphs and diagrams. The basic reproduction number (also known as basic reproduction ratio (RO) or rate) is an epidemiological metric used to measure the transmissibility of infectious agents [6]. It represents, on average, the number of people that a single infected person can be expected to transmit that infection to and thus indicates how contagious the infection is. This number helps to understand the transmission dynamics of the infection, which in turn helps the local and national authorities deploy public health measures to curb the potential spread of the disease. The value of R0 mostly depends on three factors, namely the period of infectiousness, the mode of transmission, and the contact rate (referring to the number of people exposed to an infected individual) [7]. For the calculation of R0 in our study, we used the susceptible, infectious, and recovered (SIR) epidemic model as proposed by van den Driessche [8].

Ethical considerations

The ethical clearance for this investigation was granted by the Institute Ethics Committee, All India Institute of Medical Sciences, New Delhi, India. Anonymity was maintained under all circumstances and confidentiality of all cases was ensured in the outbreak investigation.

## Results

Contact tracing could identify a total of 97 individuals who were exposed to COVID-19 cases in the context of the present outbreak, and a total of 27 individuals tested positive. These 27 cases of the outbreak included both patients (18; 66%) and HCP (9; 33%) as described in Table 1.

**Table 1:** Category of cases with a probable incubation period P: Patient, NO: Nursing officer, D: Doctor, OTA: Operation theatre assistant

Serial number	Category	Days from suspected exposure to positivity	Source
1	P1 (index case)		Community
2	OTA	2	P1
3	P2	4	OTA
4	P3	6	OTA
5	P4	7	P3
6	NO1	8	P4
7	P5	7	NO1
8	P6	5	P5
9	P7	5	P5
10	P8	6	P5
11	P9	6	P5
12	NO2	5	P5
13	P10	5	NO2
14	P11	5	NO2
15	P12	5	NO2
16	P13	5	NO2
17	NO3	4	P12
18	NO4	5	P12
19	NO5	7	P12
20	P14	5	NO4
21	P15	4	NO5
22	P16	5	NO5
23	P17	4	NO4
24	P18	5	NO5
25	D1	5	P17
26	NO6	4	P17
27	NO7	4	P17

The epidemiological link was established based on the timing of exposure, the appearance of symptoms, and laboratory confirmation of the infection. Definite, probable, and possible hospital-acquired infections of SARS-CoV-2 were found in five, four, and nine patients, respectively. The time from admission to a positive test for SARS-CoV-2 for the patients is depicted in Table 2.

**Table 2:** Patients with their source of acquisition of infection P: Patient

Patients	Number of days from admission to positivity	Nosocomial spread
P1	3	Possible, Suspected at admission
P2	15	Definite
P3	6	Possible
P4	2	Possible
P5	23	Definite
P6	18	Definite
P7	13	Probable
P8	12	Probable
P9	2	Possible
P10	6	Possible
P11	5	Possible
P12	16	Definite
P13	50	Definite
P14	3	Possible
P15	8	Probable
P16	8	Probable
P17	5	Possible
P18	2	Possible

**Figure 2:**
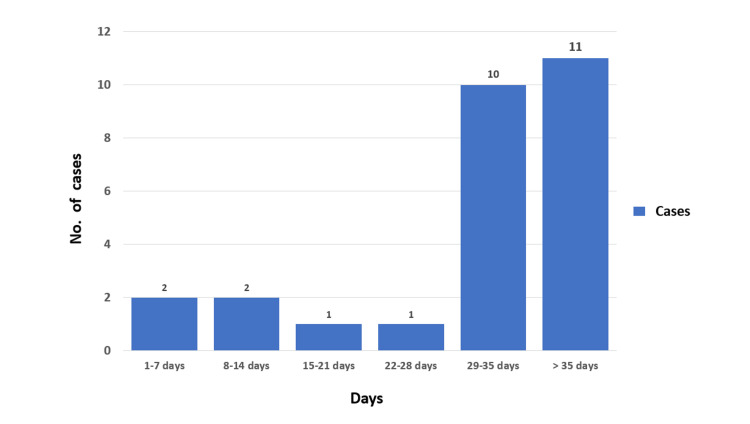
Histogram depicting the number of cases plotted against the days since the onset of the outbreak

The first laboratory-confirmed case (index case) of COVID-19 was a patient (P1) admitted to the D2 ward. A histogram with the number of cases plotted against days since the onset of the outbreak is depicted in Figure 2.

The time to detection of the index case was three days after admission. The index case had bilateral infiltrates in the chest X-ray, which raised the suspicion of COVID-19 in the setting of an ongoing pandemic, and rRT-PCR testing for SARS-CoV-2 was sought for the patient. The possible source of infection was considered to be community-acquired as the patient was symptomatic during admission. However, based on a single negative RT-PCR report, the patient was admitted to the General Medicine ward and later turned out to be positive after three days.

The second case was an operation theatre assistant (OTA) who was involved in setting up ventilators and providing care to the index case without a face shield. He became symptomatic two days after the exposure but continued to work while being symptomatic. After being advised by senior colleagues, he underwent rRT-PCR testing for SARS-CoV-2 and was found to be positive on the fifth day of exposure with the index case.

The third (P2) and fourth (P3) cases were two patients admitted to the ward who had contracted the infection from the OTA who provided care to P2 and P3 patients. The fourth case (P3) in turn infected the fifth case (P4) in the ward. The sixth case was a nursing officer (NO1) who tested positive 13 days after the index case. She was not exposed to the index case but provided care to the fifth case (P4) from whom she contracted the infection.

**Figure 3:**
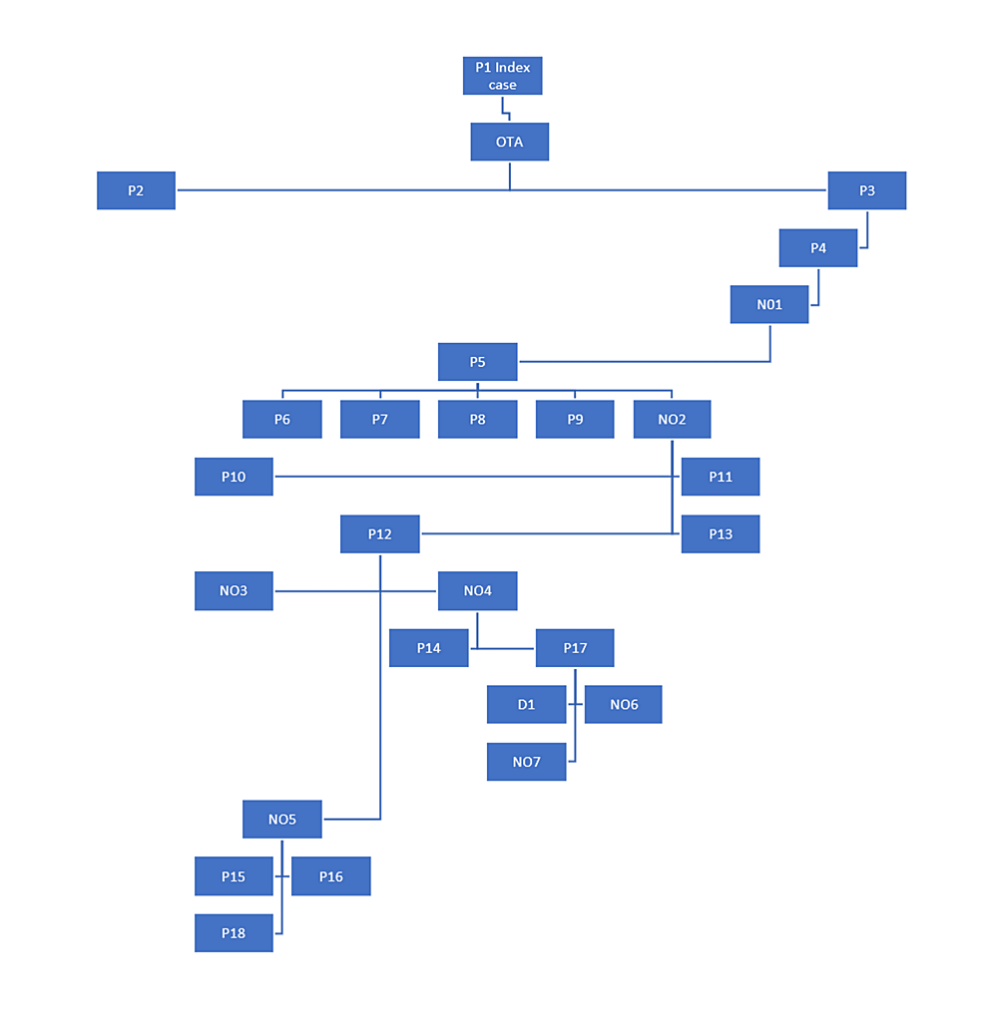
Epidemiological link of the outbreak P: Patient, OTA: Operation theatre assistant, NO: Nursing officer, D: Doctor

The cluster of cases came to the notice of the investigators when the seventh case (P5) tested positive 18 days after the index case had tested positive. The patient was not a suspect at the time of admission and initially tested negative for SARS-CoV-2 at the time of admission. He had no contact with the index case, but he was cared for by the sixth case (NO1). Subsequently, multiple patients and nursing officers tested positive. The possible epidemiological link between the cases is depicted in Figure 3.

The average incubation period of the virus in this outbreak was 5.11 days. The average R0 was 3.18, which is higher than that described in the community settings of India [9].

## Discussion

Periodic outbreaks of SARS-CoV-2 are expected with newer variants emerging periodically. In the pre-COVID era, the medical wards of our hospital were divided into cubicles/bays and each cubicle accommodated five patient beds. The distance between the two beds was 1.5 feet. The patients admitted were both critically ill and on ventilators, as well as stable patients. Since the beginning of the COVID-19 pandemic in 2020, the institutional policy has been to admit COVID-19 suspect patients in a separate COVID-19 ward, and shift them to respective specialty wards upon a negative test result. In the present outbreak, the first/index case was a patient with severe acute respiratory illness (SARI) defined according to WHO as an "individual presenting with acute respiratory infection with fever ≥ 38◦C and cough with onset within the last 10 days and requires hospitalization" [10]. She was admitted to the medicine ward after a single negative test report for COVID-19. However, since the clinical suspicion was high, a repeat test was performed, which turned out to be positive. The OTA who managed the ventilator for this patient was the second case of the outbreak. He contracted the disease two days after exposure. But while being symptomatic, he continued to work in the wards. He was the source of infection in the third (P2) and fourth cases (P3) in the ward. Patient 3 infected the fifth case (P4) who in turn infected the sixth case, a nursing officer (NO1). Nursing officer 1 infected the seventh case (P5) who in turn infected four other patients (P6 to P9) as they were in the same cubicle, and NO2 who had cared for patient P5. Nursing officer 2 infected four patients (P10 to P13). Patient 12 infected three nursing officers (NO3 to NO5). Nursing officer 4 infected two more patients: P14 and P17. Nursing officer 5 infected three more patients: P15, P16, and P18. Patient 17 infected one doctor (D1) and two more nursing officers (NO6, NO7) who were the last cases of the outbreak.

A quick response from the hospital infection control committee led to rapid control of the outbreak. Several measures were adopted. Firstly, all positive patients were immediately shifted to designated COVID-19 care areas. Secondly, the entire ward and cubicles were thoroughly cleaned and disinfected before admitting new patients in those areas. Thirdly, a maximum of four patients were allowed in each cubicle to allow more spacing between patients. Before the outbreak, each cubicle accommodated five patients. Fourthly, screening for symptoms and SARS-CoV-2 testing were arranged for attendants. Fifthly, all healthcare workers in the medical ward had to undergo laboratory testing for SARS-CoV-2. Finally, contact tracing and a mandatory 14-day quarantine of all high-risk contacts were carried out. Several factors were identified as flaws that probably led to the outbreak, viz., more patients in a single cubicle, allowing symptomatic HCP to continue work, and sub-optimal use of personal protective equipment (PPE) by HCP. The contributing factor behind these was a significantly high patient load in our tertiary care centre. The COVID-19 suspect ward was in constant need of making beds available for new suspects coming from the screening area. The scenario was not different in other tertiary care centres in the country. The HCP who contracted the disease were not allowed any special leave due to the overt crisis, and most of them preferred to suppress symptoms. The closed indoor space with centralized air conditioning and proximity between each patient bed further potentiated the spread of this highly transmissible virus.

Healthcare personnel form a connection between healthcare settings and the community, and given the essential nature of their job, they are not confined, and thus they may play a role in starting or amplifying outbreaks in settings such as hospitals [11]. Similar to our study, one case series from the United Kingdom concluded that SARS-CoV-2 infection might be introduced to an area in the hospital by HCP who were asymptomatic or minimally symptomatic. Not only staff members, but asymptomatic patients and their attendants might also act as carriers of COVID-19, carrying the infection into and around the hospital and leading to unexpected transmission events. The fact that the transmission of infection can occur from pre-symptomatic, asymptomatic, and minimally symptomatic individuals to others also highlights the importance of stringent measures to prevent transmission in the hospital setting [12]. In a study in one of the tertiary care hospitals in central Paris, France, it was seen that residual transmissions of infection occurred due to persistent exposures with undiagnosed patients or colleagues, and universal masking, hand hygiene, PPE, and medical masks for patients not only allowed protection of HCP but also helped in the containment of the outbreak [13]. There was a similar scenario in the outbreak at our hospital setting. An HCP who came into direct contact with patients with COVID-19 got infected, especially when universal masking was not strictly followed. The same was evident in an online assessment of employees from eight hospitals in southeast Michigan. The report revealed that employees who came into direct contact with patients with COVID-19 were more likely to be seropositive, but in participants who wore masks during exposure to COVID-19, the likelihood of seropositivity was much less. Also, a large proportion of seropositive employees self-reported as being asymptomatic [14]. 

The literature on SARS-CoV-2 transmission suggests that the risk of transmission depends on several factors, which include contact pattern, host-related infectivity/susceptibility pattern, environment, and also socioeconomic factors [15]. In our setting, too, a few factors led to the outbreak. These underscored the importance of several fronts where we were required to improve to contain the outbreak and prevent any future outbreaks. Similar findings emerged from another outbreak of COVID-19 after an on-site church service in a nursing home in the Netherlands. The investigation elucidated the sources and chain(s) of transmission. A thorough scrutiny revealed a complex picture of viral transmission, which indicated widespread regional circulation of the virus in the weeks before the outbreak and also showed the efficacy of stringent measures to control transmission, especially if they were implemented at an early stage [16].

The present outbreak investigation had a few limitations. Firstly, this was an investigational study and not a case-control study. For this reason, it could not directly identify the different risk factors contributing to SARS-CoV-2 infections. Secondly, genomic analysis of the SARS-CoV-2 identified in different cases was beyond the scope of the present study. Therefore, the genetic relatedness of the outbreak strains of the virus could not be proven. Thirdly, the quantitative microbial risk assessment (QMRA) for this outbreak was not done due to the limited time to achieve quick control of the outbreak.

## Conclusions

Severe acute respiratory syndrome coronavirus 2 is a highly transmissible virus, and the transmissibility is even higher in hospital settings with closed ventilation and greater proximity between susceptible patients. Designating COVID-19 suspect patients as negative based on a single negative rRT-PCR for SARS-CoV-2 may be responsible for an outbreak or clustering of cases in hospital settings. The continued work in the ward by exposed HCP who are minimally symptomatic or asymptomatic is another reason to be considered. A patient with a high index of suspicion should be treated as positive, irrespective of test results. Periodic testing of all HCP irrespective of symptoms, should be done. The importance of COVID-19-appropriate behavior in hospitals which includes universal masking, hand hygiene, physical distancing, and the appropriate use of PPE, cannot be overemphasized. Although the WHO has announced that the COVID-19 pandemic is over, the threat of a rapid surge of cases still exists due to the emergence of new variants of SARS-CoV2. The present investigation, therefore, would be deemed relevant for many years to come. Furthermore, the measures that have been mentioned for the containment of the outbreak might be of immense help in initiating a rapid response against any potential outbreaks of a similar kind in the near future.
